# Accelerated Post-contrast Wave-CAIPI T1 SPACE Achieves Equivalent Diagnostic Performance Compared With Standard T1 SPACE for the Detection of Brain Metastases in Clinical 3T MRI

**DOI:** 10.3389/fneur.2020.587327

**Published:** 2020-10-27

**Authors:** Augusto Lio M. Goncalves Filho, John Conklin, Maria Gabriela F. Longo, Stephen F. Cauley, Daniel Polak, Wei Liu, Daniel N. Splitthoff, Wei-Ching Lo, John E. Kirsch, Kawin Setsompop, Pamela W. Schaefer, Susie Y. Huang, Otto Rapalino

**Affiliations:** ^1^Department of Radiology, Massachusetts General Hospital, Boston, MA, United States; ^2^Athinoula A. Martinos Center for Biomedical Imaging, Charlestown, MA, United States; ^3^Harvard Medical School, Boston, MA, United States; ^4^Siemens Healthcare GmbH, Erlangen, Germany; ^5^Siemens Shenzhen Magnetic Resonance Ltd., Shenzhen, China; ^6^Siemens Medical Solutions, Boston, MA, United States; ^7^Harvard-MIT Division of Health Sciences and Technology, Cambridge, MA, United States

**Keywords:** brain, metastases, magnetic resonance imaging, parallel imaging, Wave-CAIPI, post-contrast, high-resolution, 3D

## Abstract

**Background and Purpose:** Brain magnetic resonance imaging (MRI) examinations using high-resolution 3D post-contrast sequences offer increased sensitivity for the detection of metastases in the central nervous system but are usually long exams. We evaluated whether the diagnostic performance of a highly accelerated Wave-controlled aliasing in parallel imaging (Wave-CAIPI) post-contrast 3D T1 SPACE sequence was non-inferior to the standard high-resolution 3D T1 SPACE sequence for the evaluation of brain metastases.

**Materials and Methods:** Thirty-three patients undergoing evaluation for brain metastases were prospectively evaluated with a standard post-contrast 3D T1 SPACE sequence and an optimized Wave-CAIPI 3D T1 SPACE sequence, which was three times faster than the standard sequence. Two blinded neuroradiologists performed a head-to-head comparison to evaluate the visualization of pathology, perception of artifacts, and the overall diagnostic quality. Wave–CAIPI post-contrast T1 SPACE was tested for non-inferiority relative to standard T1 SPACE using a 15% non-inferiority margin.

**Results:** Wave–CAIPI post-contrast T1 SPACE was non-inferior to the standard T1 SPACE for visualization of enhancing lesions (*P* < 0.01) and offered equivalent diagnostic quality performance and only marginally higher background noise compared to the standard sequence.

**Conclusions:** Our findings suggest that Wave-CAIPI post-contrast T1 SPACE provides equivalent visualization of pathology and overall diagnostic quality with three times reduced scan time compared to the standard 3D T1 SPACE.

## Introduction

Brain metastases are the most common tumors of the central nervous system (CNS) (1). However, their true incidence is probably underestimated as they may be asymptomatic in 60–70% of cases (2), or even some are overlooked in severely ill patients. Nevertheless, the current advancements in immunotherapeutic agents and improved local stereotactic radiosurgery demonstrate the importance of early surveillance for brain metastases. If CNS metastases are recognized earlier, when patients still have a good performance status, they can benefit from more aggressive treatment strategies (3). Certain malignancies are more frequently associated with brain metastases, including cancers of the lung, breast, skin (melanoma), colon, kidney, pancreas, testes, ovary, and cervix (1, 2, 4). Moreover, melanomas have the highest preference to metastasize to the brain (~50%) (5).

Brain magnetic resonance imaging (MRI) provides high sensitivity for non-invasive diagnosis of intracranial metastases. It allows for a detailed evaluation of the different compartments of the CNS, including the skull, brain parenchyma, ependymal surface, leptomeninges, and pachymeninges. The intrinsic superior soft-tissue contrast and the multiplanar capability of MR imaging increase the sensitivity for the screening of secondary tumor implants. Metastases typically enhance after administering gadolinium contrast material due to the absence of the blood-tumor barrier (6). Contrast-enhanced MRI is considered the preferred modality for the evaluation of metastatic disease and is superior to other modalities such as computed tomography in detecting metastases from systemic melanoma or breast cancer (6).

There is much debate regarding which post-contrast T1-weighted pulse sequence is the best. The preference may vary according to the available field strength and other limitations in hardware and software resources at different sites. 3 Tesla (3T) MRI offers better signal-to-noise ratio and produces higher contrast between tumor and normal brain tissue than at 1.5T (7). Magnetization prepared 3D gradient recalled echo pulse sequences, that include MPRAGE, IR-SPGR, and BRAVO, are T1-weighted sequences that show excellent anatomical depiction and are widely available in clinical protocols. In fact, they are considered a pillar sequence in the standardized brain tumor protocol for gliomas (8) and in the minimum requirements for brain imaging recommended by the Response Assessment in Neuro-Oncology–Brain Metastases working group (9). However, gradient recalled echo (GRE)-based pulse sequences show brighter white matter signal and may potentially diminish the conspicuity of enhancing lesions due to the reduced contrast ratio (5). Conversely, Spin-Echo (SE)-based sequences offer increased contrast in enhancing lesions and better flow suppression, facilitating the distinction of cortical subcentimeter enhancing metastases from vessels that might otherwise appear as bright dots in GRE-based sequences (5).

The introduction of optimized 3D fast/turbo SE imaging, such as sampling perfection with application-optimized contrasts using different flip angle evolutions (SPACE), offers a robust and flexible approach for 3D SE-based imaging with the benefits of optimal contrast depiction and the added advantage of multiplanar reformatted viewing for evaluating tumor within the complex brain anatomy (10). A meta-analysis by Suh et al. (11) included studies that compared the detectability of brain metastases using SE or GRE contrast-enhanced sequences and found that 3D SE images using 1 mm thick slices are preferred for detecting brain metastases in 3T scans, notably for the detection of small lesions (11). Thus, the ideal recommended contrast-enhanced pulse sequence suggested in the most recent consensus publication on a standardized protocol for brain metastases imaging (5) favors the 3D SE-based sequence (SPACE) over the GRE-based pulse sequence (MPRAGE).

Several advanced MR techniques, including proton MR spectroscopy, diffusion, and perfusion imaging, increase the precision of tumor characterization and support the distinction of metastases from other entities. Hence, MR brain protocols for the evaluation of neoplasms often consist of multiple standard and advanced sequences that result in a prolonged scanning time, which may contribute to motion artifacts (12) and patient anxiety (13). The introduction of a new encoding technology that can accelerate the scan time of high-resolution sequences could facilitate broader application of the advantages of 3D SE-based MRI, such as SPACE (14). Wave-controlled aliasing in parallel imaging (Wave-CAIPI) is an advanced technique that combines a corkscrew gradient trajectory with CAIPI shifts in the ky and kz directions to efficiently encode k-space and evenly spread the voxel aliasing in all dimensions, taking full advantage of the 3D coil sensitivity information to provide highly accelerated parallel imaging with negligible artifact and signal-to-noise ratio penalties (15). Wave-CAIPI is an advanced parallel imaging encoding technique that has been demonstrated to achieve highly accelerated structural imaging with negligible noise amplification using standard scanner hardware (16).

The goal of this study was to compare a highly accelerated Wave-CAIPI post-contrast 3D T1 SPACE sequence (Wave-T1 SPACE) with the commonly used standard high-resolution 3D T1 SPACE sequence for routine clinical brain imaging at 3T. We hypothesized that Wave-T1 SPACE is non-inferior to the standard sequence with equivalent diagnostic quality and an estimated three-fold reduction of acquisition time.

## Method

### Selection of Participants and Study Design

With Institutional Review Board (IRB) approval, 33 patients undergoing clinical brain MRI with and without contrast for the evaluation of brain metastases at a single institution were consecutively enrolled. Adult patients (age > 18 years) were scanned on a 3T MRI scanner (MAGNETOM Prisma, Siemens Healthcare, Erlangen, Germany) using a commercially available 20- or 32-channel receiver coil array. The study was Health Insurance Portability and Accountability Act compliant. The need for informed consent was waived by the institution's IRB since all MRI exams were acquired as part of the standard care of the enrolled individuals, without significant added time to each exam (i.e., <2 min of additional imaging per case). Instead, patients were provided with an information sheet describing the scope of the research study and could opt out prior to the start of the scan. All participants had a prior confirmed diagnosis of systemic tumor and came for MRI evaluation, in both inpatient and outpatient settings, in search of intracranial metastases or to evaluate previously diagnosed metastatic disease. Distribution of the study subjects and detailed systemic oncologic diagnoses are demonstrated in Table 1.

**Table 1:** Demographic information and clinical diagnoses of participants.

**Oncologic cases (*N* = 33)**	
Age (mean ± SD, year)	58.2 ± 13.5
Sex (%)	
Male	12 (36%)
Female	21 (64%)
Systemic diagnosis (%)	
Melanoma	12 (36%)
Lung cancer	8 (24%)
Gastrointestinal cancer	5 (15%)
Breast cancer	4 (12%)
Lymphoma	1 (3%)
Thyroid cancer	1 (3%)
Biliary cancer	1 (3%)
Sarcoma	1 (3%)

### MRI Protocol

The accelerated post-contrast Wave-T1 SPACE was embedded in the standard contrast enhanced brain MRI protocol for oncologic evaluation. Each scan included a standard post-contrast T1 SPACE sequence and Wave-T1 SPACE sequence. Gadolinium-enhanced images were obtained after intravenous administration of standard dose of 0.2 ml/kg (0.1 mmol/kg) of gadoterate meglumine (Dotarem®, Guerbet; Paris, France) at a flow rate of ~2 ml/s. Twenty-four studies were performed with the standard post-contrast T1 SPACE sequence acquired before Wave-T1 SPACE, and 9 studies were performed with the sequence order inverted, acquiring Wave-T1 SPACE before the standard T1 SPACE, to control for potential differences related to the order of acquisition.

### Wave-CAIPI Post-contrast T1 SPACE Sequence and Reconstruction

Wave-T1 SPACE was implemented using a prototype single slab 3D fast spin echo SPACE sequence (15). On-line reconstruction was performed using an autocalibrated procedure in which the true gradient trajectory is estimated during the reconstruction without the need for additional calibration scans. This allowed for simultaneous estimation of the parallel imaging reconstruction and the true k-space trajectory (17), with a reconstruction time of ~60 s. We matched the main pulse sequence parameters that contribute to T1-weighted contrast (i.e., TR, TE, and flip angle) between the Wave-T1 SPACE and standard T1 SPACE sequences. The standard T1-SPACE sequence used in our institution's routine clinical protocol employs the default vendor reconstruction filter, which introduces a small degree of spatial smoothing. To provide comparable effective spatial resolution using the prototype Wave-T1 SPACE sequence, a slightly larger isotropic voxel size was used (0.9 vs. 1.0 mm). This resulted in visually comparable effective spatial resolution as evaluated by the study neuroradiologists. Additional sequence parameters are shown in Table 2.

**Table 2:** Pulse sequence acquisition parameters.

	**Standard T1-SPACE**	**Wave-T1 SPACE**
**Acquisition parameters**		
FOV read (mm)	230	256
Matrix size	256 × 256	256 × 256
Slice thickness (mm)	0.9	1.0
TR/TE (ms)	700/11	700/12
Flip angle (degree)	120	120
Echo train length	38	43
Acceleration factor *R*	GRAPPA, *R* = 4	Wave-CAIPI, *R* = 9
Scan time	4 min 19 s	1 min 40 s

### Image Evaluation

Two neuroradiologists (O.R and S.Y.H.) with 18 and 8 years of experience, respectively, independently reviewed all images in a blinded and randomized fashion. A pre-determined 5-point grading scale was used to compare Wave-T1 SPACE with the standard T1 SPACE, following the scales set for previously published clinical validation studies of Wave-CAIPI sequences (18) and adapted for the evaluation of enhancing lesions. After the DICOM datasets had been anonymized, reviewers evaluated only the post-contrast images on an independent workstation and were allowed to adjust the window width and level settings for each image series for optimal viewing.

Reviewers underwent several head-to-head analysis sessions in which they evaluated the detection of pathological enhancement in common locations for metastatic seeding (parenchymal, leptomeningeal, dural, and ependymal), the presence of artifacts related to motion or background image noise, and the overall diagnostic quality. The screen position of the sequences and the order of the cases were randomized.

All cases were rated for each feature within the 5-point grading scale, where positive numbers favored the sequence on the right and negative numbers favored the sequence on the left side of the screen (Supplementary Table 1). Disagreements between readers were adjudicated by a third neuroradiologist (P.W.S.) with over 20 years of experience.

### Statistical Analysis

We tested for non-inferiority of Wave-T1 SPACE compared to standard T1 SPACE in the head-to-head analysis. A non-inferiority margin (Δ) of 15% was chosen as part of a larger, systematic evaluation of Wave-T1 SPACE for post-contrast imaging, with the null hypothesis (H_0_) that the proportion of cases where standard T1 SPACE was preferred over Wave-T1 SPACE was >15% (19). We used the Z statistic to calculate the probability of the standard sequence being preferred over the Wave-T1 SPACE sequence in more than 15% of cases (H_0_ > Δ), with a type 1 error rate (α) of 0.05. Other descriptive data were summarized by the calculation of means and standard deviations. We also calculated the upper bound of the 95% confidence interval for the proportion of cases where standard T1 SPACE was preferred over Wave-T1 SPACE, i.e., the critical value, *P*_critical_. The interrater agreement was reported using the quadratically weighted Cohen κ to disproportionately penalize larger disagreements. The agreement of categorical variables was interpreted according to Landis and Koch (20). All statistical calculations were performed using R version 3.6.3.

## Results

**Figure 1:**
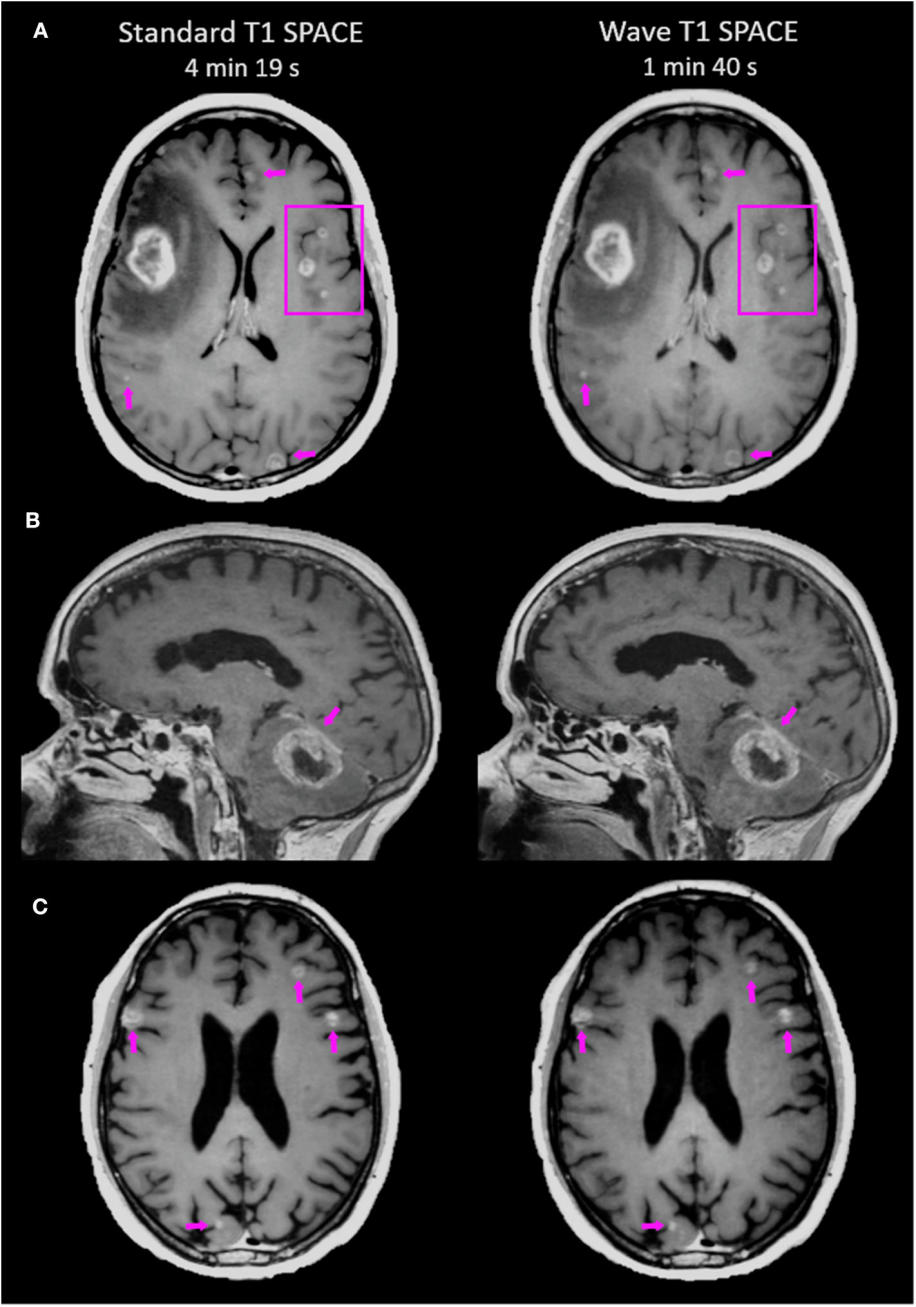
Representative images comparing the post-contrast Standard T1 SPACE and Wave-T1 SPACE sequences. **(A)** A 25-year-old female with metastatic melanoma presenting a large mass in the right frontal lobe. Other smaller scattered enhancing metastases are visualized in both hemispheres (arrows and box). **(B)** Infratentorial intraparenchymal metastasis in a 76-year-old female with a history of melanoma. There is also abnormal dural enhancement on the overlying tentorium (arrow). **(C)** Multiple cortical/subcortical metastases in a 54-year-old man with lung cancer are equally visualized in both sequences (arrows).

**Figure 2:**
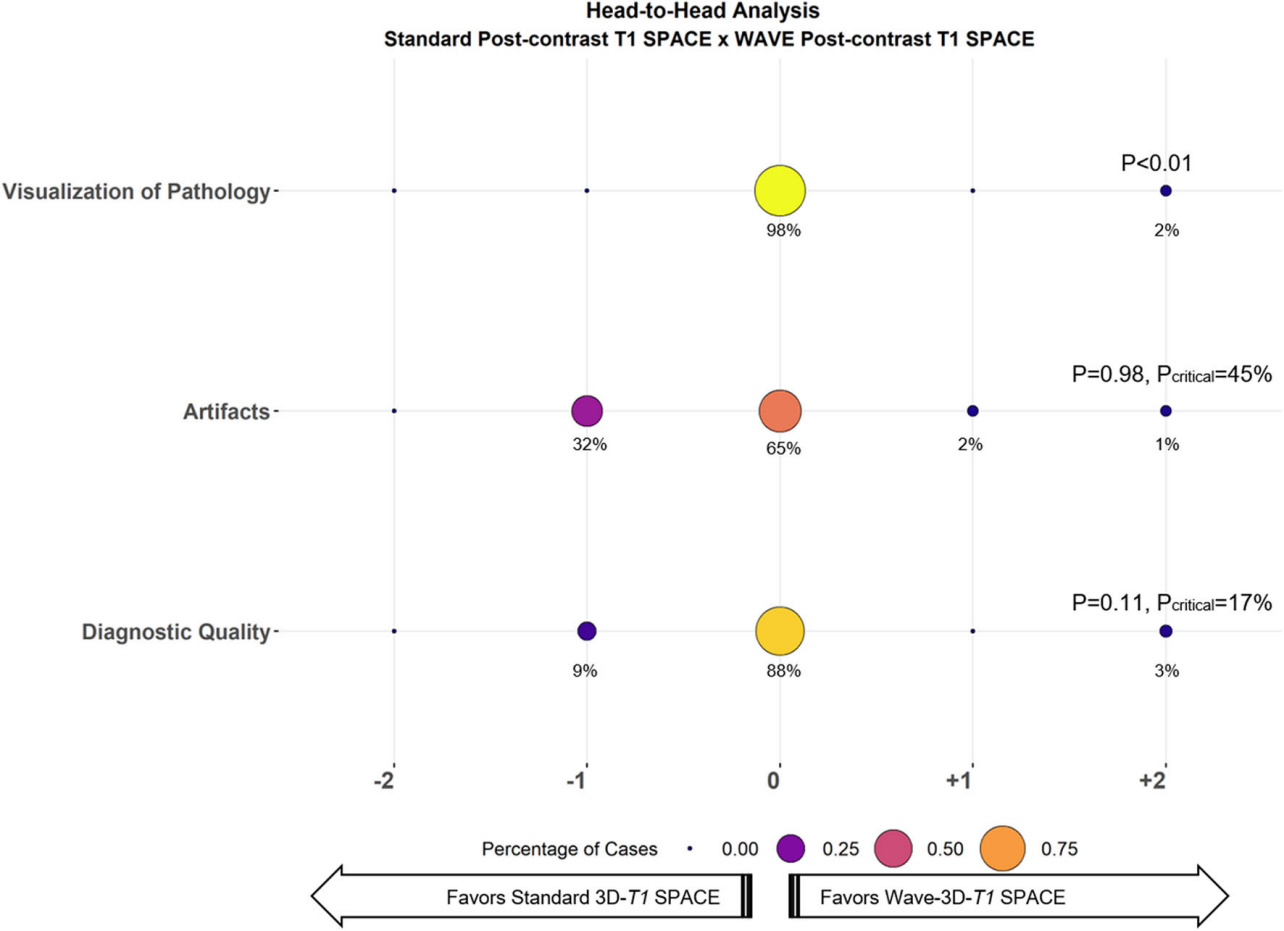
Balloon plot showing the results of the head-to-head comparison of Standard T1 SPACE and Wave-T1 SPACE for visualization of pathology (i.e., enhancing lesions), artifacts, and diagnostic quality. Each circle's size and color represent the percentage of cases assigned a given score from a total of 33 cases. The percentage of cases receiving a given score is indicated below each circle. A zero-score indicates equivalency, negative scores (left) favor Standard T1 SPACE, and positive scores (right) favor Wave-T1 SPACE. The critical value (*P*_critical_) is also provided, corresponding to the upper bound of the 95% confidence interval for the proportion of cases in which Standard T1 SPACE was preferred.

All the 33 oncologic cases were successfully acquired and evaluated. In the head-to-head comparison, abnormal enhancement concerning for metastases was detected in 20 cases (60%) (Figure 1). Of the 20 cases that showed abnormal enhancement, 15 (75%) had parenchymal enhancement, 10 (50%) had dural enhancement, 10 (50%) had leptomeningeal enhancement, and 2 (10%) had ependymal enhancement, with 11 showing more than one type of enhancing lesion. Interrater agreement ranged from moderate to substantial (κ = 0.40 for visualization of enhancing lesions, 0.52 for artifacts, 0.68 for diagnostic quality). The results of the head-to-head comparison and the non-inferiority testing are shown in Figure 2. Wave-T1 SPACE was non-inferior to standard T1 SPACE for delineating enhancing pathology with most cases being rated as equivalent by reviewers (19 of 20 cases, 98%). In one case (1 of 20 cases, 2%), Wave-T1 SPACE was preferred over the standard sequence. From the complete cohort, most cases (65%) were considered equivalent for evaluation of the general perception of artifacts, in 18 cases (55%) for noise level and 25 cases (76%) for motion. In only two cases (3%), the Wave-T1 SPACE was preferred over the standard sequence for perception of artifacts. The standard sequence was preferred over Wave-T1 SPACE for presenting less background noise in 14 cases (42%) and in 7 cases (21%) for fewer motion artifacts, but this difference did not affect the visualization of underlying structures nor obscured any small lesions. The overall diagnostic quality was considered equivalent in 88% of the sample (29 cases, of 33). In three cases (9%), the standard sequence was preferred without affecting the final clinical diagnosis. In one case (3%), Wave-T1 SPACE was preferred to the extent that it would affect the final clinical diagnosis, due to the presence of extensive motion artifact on the standard sequence. To better validate the comparison of Wave-T1 SPACE with the standard sequence, we also performed additional sub analyses by coil channel, by order of acquisition, and expanded the evaluation of visualization of enhancement by each compartment (parenchymal, leptomeningeal, dural, and ependymal). The results showed that Wave-T1 SPACE provided equivalent visualization of enhancing pathology independently of the number of channels in the coil array and the order of acquisition (Supplementary Figures 1–5). However, given the larger number of independent coil elements in the 32-channel coil, we observed an improvement in the image signal-to-noise ratio (SNR) and noise amplification (g-factor), which translated in a better performance of the diagnostic quality in this subset of cases.

## Discussion

This study compared the performance of highly accelerated 3D Wave-T1 SPACE to the standard 3D T1 SPACE sequence in the visualization and diagnostic evaluation of brain metastases. Wave-T1 SPACE showed equivalent diagnostic performance for delineating enhancing metastases and was three times faster than the standard sequence. Wave-T1 SPACE images were slightly noisier compared to the standard sequence, but this difference did not interfere with the final diagnosis. Our findings suggest that Wave-T1 SPACE could replace standard T1 SPACE for the evaluation of brain metastases, as the advantages of lesion detection of the thin slice 3D SE-based pulse sequence are preserved and the gains in saved acquisition time would improve patient comfort and utilization of MR resources. The decreased scan time of Wave-CAIPI may overcome the slight underperformance in image quality from slightly greater noise, likely due to the high intrinsic contrast-to-noise ratio of the 3D SE-based pulse sequence for enhancing lesions.

The savings in acquisition time without loss of clinically important information can provide synergistic benefits with the combined use of accelerated sequences that shorten the overall exam time and may improve utilization of MR resources, particularly in motion-prone populations. The Wave-CAIPI encoding approach has been applied to other imaging sequences providing complementary contrasts such as susceptibility weighted imaging (18, 21) and structural MPRAGE (without IV contrast administration) (22). Combining multiple Wave-CAIPI based 3D acquisitions could synergistically further reduce acquisition times and increase patient throughput, to the benefit of the patients and their providers.

Our study has several limitations. First, we have a relatively small sample size due to the proof-of-concept design within the specific indication of contrast-enhanced imaging for the evaluation of brain metastases. Other relevant limitations involve the heterogeneity of the multiple primary tumors and differences in tumor biology among participants. Nevertheless, our findings show a clear trend in the benefits of reduced scan time with preserved sensitivity for lesion detection, suggesting that these findings might be generalized to many tumor types. The small sample size probably underpowered the non-inferiority test, and the results could be considered as the basis for replicating these findings in a larger tumor-specific future study.

Second, we observed slightly greater artifacts with Wave-T1-SPACE than Standard-T1-SPACE (standard sequence preferred in 32%, Wave sequence preferred in 3%, no preference in 65%). Artifacts in 3D SE-based sequences, including SPACE, arise through a variety of mechanisms. Because it can be difficult for the radiologist to be certain of the mechanism of a given artifact, we grouped the different causes of artifact in a single category. Possible explanations for the increased artifacts observed in Wave-T1 SPACE include interactions between the Wave-CAIPI approach and motion/flow-related artifacts (possibly exacerbated by high vascular signal in the presence of gadolinium contrast), the free induction decay (10) and other 3D SE related artifacts, or imperfections in the Wave-CAIPI acquisition and reconstruction procedure itself. Although these factors did not result in the obscuration of any enhancing lesions and did not alter the radiologists' overall assessment of diagnostic quality, further evaluation of the underlying causes (and strategies for artifact mitigation) is warranted before a more general application of Wave-T1-SPACE in a clinical setting.

Third, although we did our best to balance the order of acquisition for the post-contrast standard and Wave-T1 SPACE sequences to control for potential differences in the conspicuity of enhancing lesions related to the time elapsed between contrast injection and image acquisition, more studies had standard T1 SPACE acquired before Wave-T1 SPACE (24 vs. 9). Despite this imbalance, 98% of the cases were rated as equivalent for visualization of enhancing lesions, attesting to the minimal contribution of acquisition order to the overall degree of enhancement on either sequence, as supported by the head-to-head analysis results as well as qualitative assessment of the images by all three raters.

Finally, the selection of a suitable non-inferiority margin for imaging studies is often challenging. Our selection was informed by a review of similar imaging-based non-inferiority studies and consensus among our group of neuroradiologists that the new sequence could be considered non-inferior if the standard sequence were preferred in fewer than 15% of cases. Since this threshold is essentially subjective, we also reported the critical value (*P*_critical_), equivalent to the upper bound on a 95% confidence interval for the proportion of cases in which the standard sequence was preferred. Lastly, although readers were blinded to the acquisition protocol, some aspects of the images might have allowed the readers to identify the pulse sequence being evaluated, which could introduce bias. We sought to minimize this possibility by matching the most important parameters that determine image quality and image contrast (including TR, TE, and flip angle) between acquisitions.

## Conclusion

In conclusion, we show that contrast-enhanced Wave-CAIPI 3D T1 SPACE provides equivalent visualization of enhancing lesions and overall diagnostic quality for evaluating brain metastases with three times reduction in scan time compared to standard 3D T1 SPACE. The clinical application of the Wave-CAIPI approach may facilitate more efficient utilization of MR resources without loss of clinically valuable information, which can be especially beneficial to motion-prone patients with brain metastases. The present study offers several opportunities for future study, including the mechanisms and appearance of Wave-T1 SPACE artifacts, and supports the promise and continued evaluation of post-contrast Wave-T1 SPACE for routine use in clinical practice and clinical trials.

## Data Availability Statement

The raw data supporting the conclusions of this article will be made available by the authors, without undue reservation.

## Ethics Statement

The studies involving human participants were reviewed and approved by Mass General Brigham Institutional Review Boards. Written informed consent for participation was not required for this study in accordance with the national legislation and the institutional requirements.

## Author Contributions

AG wrote the first draft of the manuscript, organized the database, and performed the statistical analysis. AG, JC, ML, PS, SH, and OR contributed to the conceptualization and design of the study, as well as to the interpretation of the data. SC, DP, WL, DS, W-CL, JK, and KS contributed to technical expertise on the acquisition and reconstruction of the data. All authors contributed to manuscript revision and review and approved the submitted version.

## Conflict of Interest

DP, WL, DS, and W-CL were employed by the company Siemens Healthineers. The remaining authors declare that the research was conducted in the absence of any commercial or financial relationships that could be construed as a potential conflict of interest. The handling editor is currently organizing a Research Topic with one of the authors SH.
