# Supplementary material for: Accelerated Post-contrast Wave-CAIPI T1 SPACE Achieves Equivalent Diagnostic Performance Compared With Standard T1 SPACE for the Detection of Brain Metastases in Clinical 3T MRI
Source: Front Neurol. 2020 Oct 27;11:587327. doi: 10.3389/fneur.2020.587327 (PMC7653188; doi:10.3389/fneur.2020.587327)
Supplement: Supplementary file 1 [file Table_1.DOCX]

Supplementary Material

**Supplementary Table.** Semiquantitative scoring criteria used for the head-to-head comparison of post-contrast Standard T1 SPACE vs. Wave-T1 SPACE.

| **Parameter** | **Favors Image A*** | |  | **Favors Image B*** | |
| --- | --- | --- | --- | --- | --- |
|  | **Score -2** | **Score -1** | **0** | **Score +1** | **Score +2** |
| **Visualization of Pathology**** | Visualization of pathology is  superior on Image A; lesions are missed on Image B | Visualization of pathology is preferred on Image A, but lesions are still visualized on Image B | Equivalent | Visualization of pathology is preferred on Image B, but lesions are still visualized on Image A | Visualization of pathology is superior on Image B; lesions are missed on Image A |
| **Artifacts** | Image B has more artifacts that may obscure small lesions | Image B has more artifacts, but small lesions are not obscured | Equivalent | Image A has more artifacts, but small lesions are not obscured | Image A has more artifacts that may obscure small lesions |
| **Overall Diagnostic Quality** | Image B is of lower quality, and the difference alters the clinical diagnosis | Image B is of lower quality, but the difference does not alter the clinical diagnosis | Equivalent | Image A is of lower quality, but the difference does not alter the clinical diagnosis | Image A is of lower quality, and the difference alters the clinical diagnosis |
| * The Wave-T1 SPACE and Standard T1 SPACE sequences were randomly positioned on either the right or left side of the screen, labeled ‘Image A’ and ‘Image B’.  ** The specific pathologies evaluated in this study were: Parenchymal, dural, leptomeningeal, and ependymal enhancement. | | | | | |
