# Supplementary material for: Accelerated Post-contrast Wave-CAIPI T1 SPACE Achieves Equivalent Diagnostic Performance Compared With Standard T1 SPACE for the Detection of Brain Metastases in Clinical 3T MRI
Source: Front Neurol. 2020 Oct 27;11:587327. doi: 10.3389/fneur.2020.587327 (PMC7653188; doi:10.3389/fneur.2020.587327)

Supplementary Material

Supplementary Figures. Balloon plots showing the results of the sub analyses of the head-to-head comparison of Standard T1 SPACE and Wave-T1 SPACE for visualization of pathology, artifacts, and diagnostic quality by the number of channels in the coil array used (Figure 1 and Figure 2), by order of acquisition (Figure 3 and Figure 4), and an expanded evaluation of visualization of enhancement by each compartment (parenchymal, leptomeningeal, dural, and ependymal), noise and motion artifacts (Figure 5). Each circle's size and color represent the percentage of cases assigned a given score. The percentage of cases receiving a given score is indicated below each circle. A zero-score indicates equivalency, negative scores (left) favor Standard T1 SPACE, and positive scores (right) favor Wave-T1 SPACE. The critical value ($P_{critical}$) is also provided, corresponding to the upper bound of the 95% confidence interval for the proportion of cases in which Standard T1 SPACE was preferred.

Figure 1. Results of sub analysis of cases performed with a 20-channel coil (N= 15).

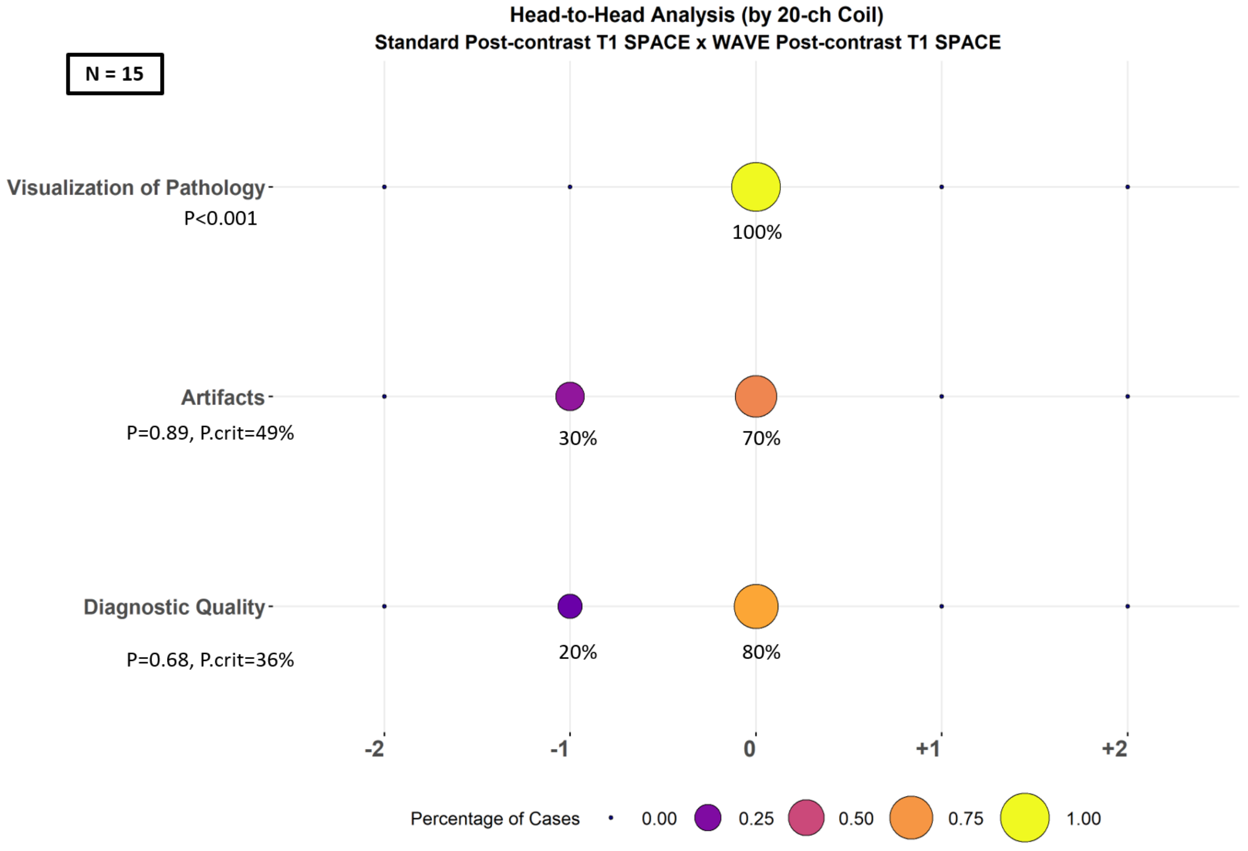

Figure 2. Results of sub analysis of cases performed with a 32-channel coil (N= 18).

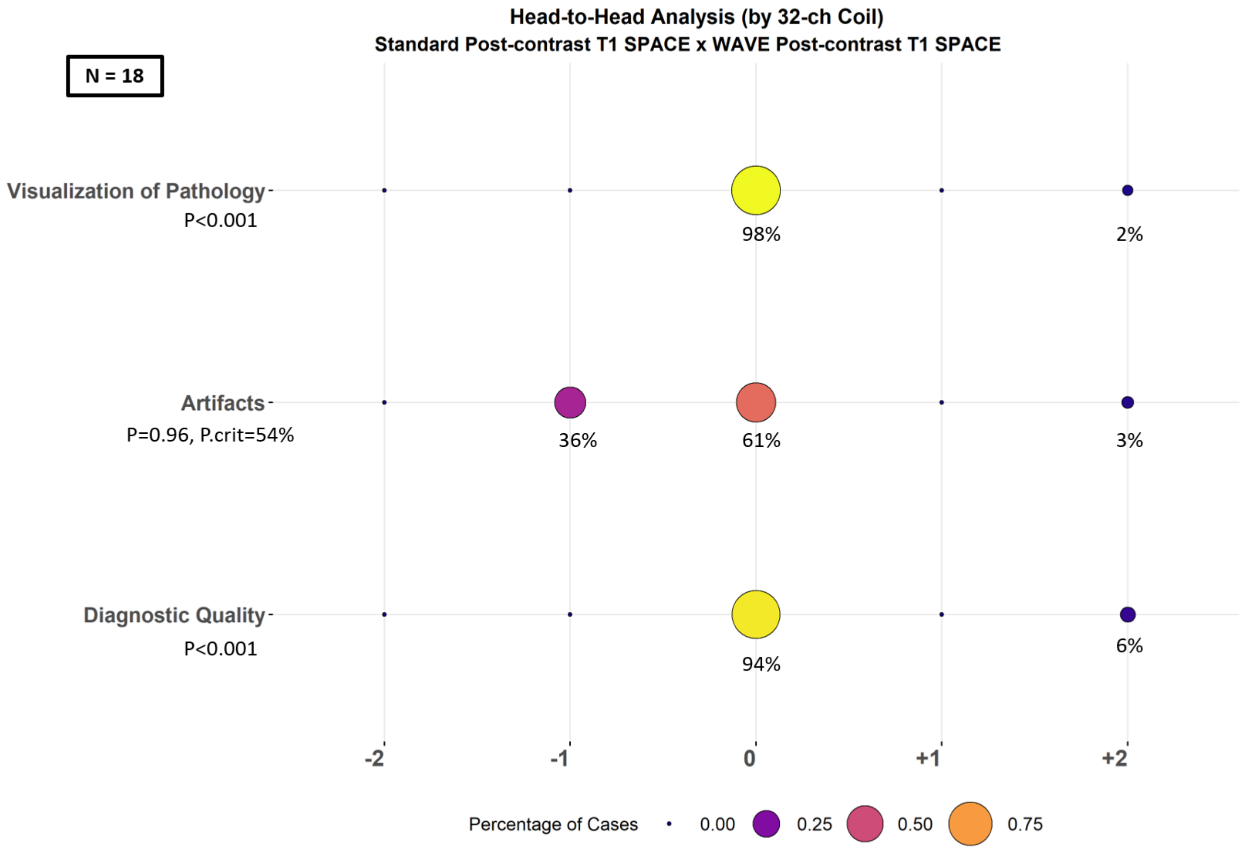

Figure 3. Results of sub analysis of cases performed with Standard T1 SPACE before Wave-T1 SPACE (N= 24).

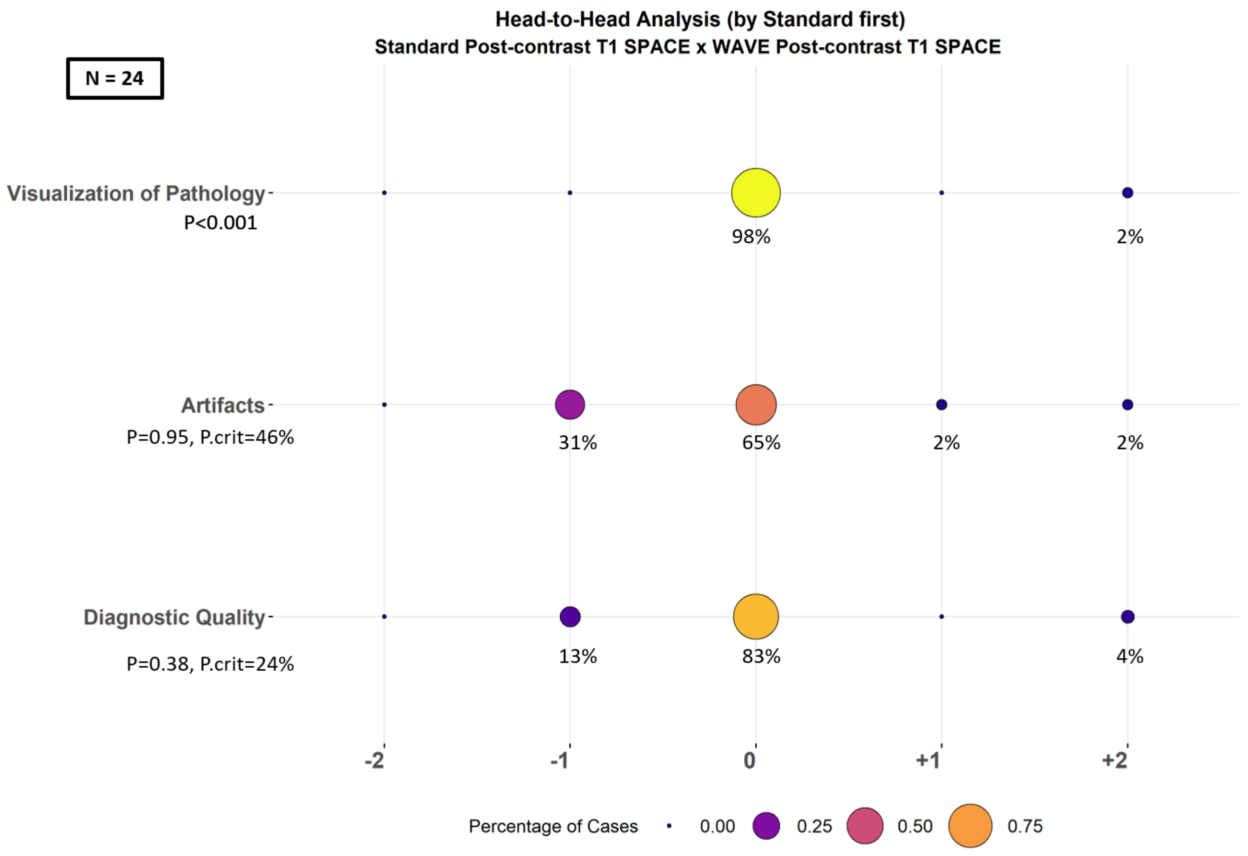

Figure 4. Results of sub analysis of cases performed with Wave-T1 SPACE before Standard-T1 SPACE (N= 9).

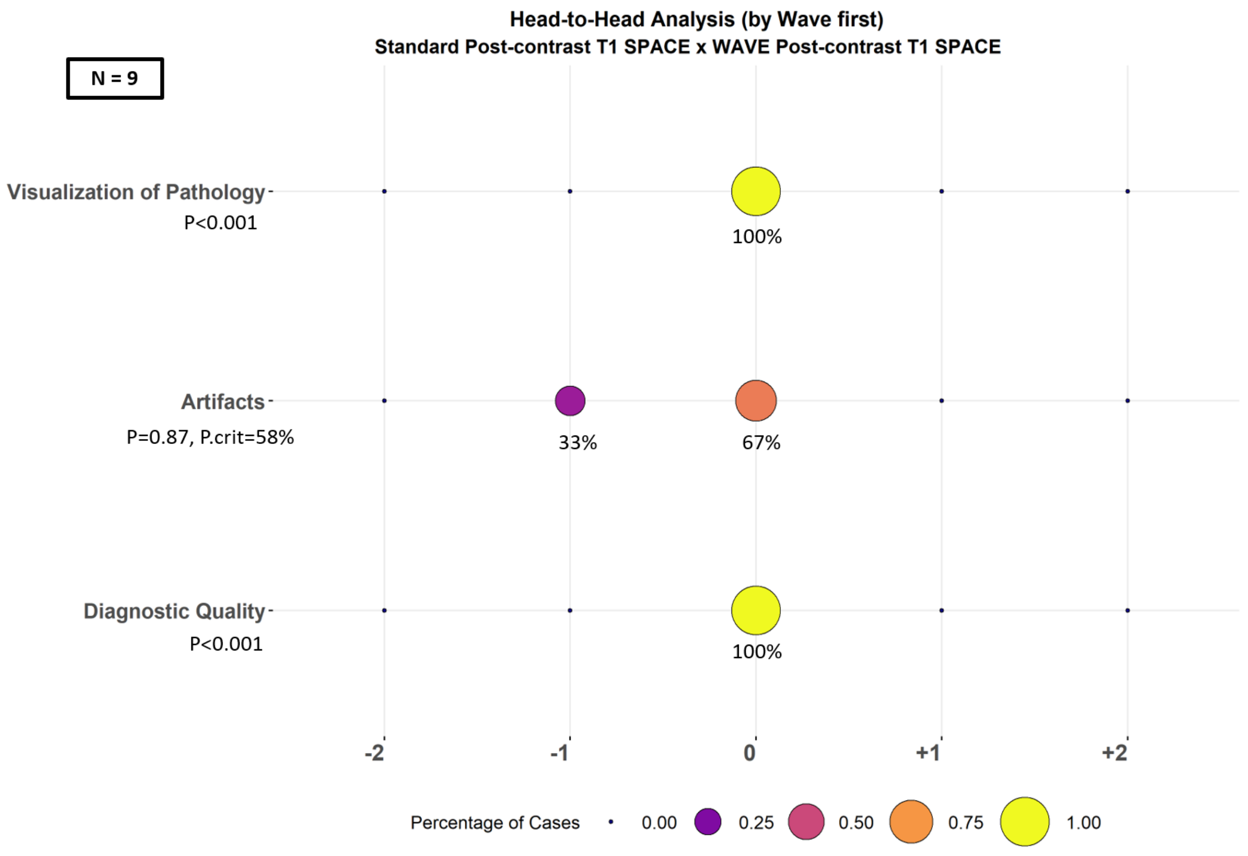

Figure 5. Results of sub analysis of cases expanded by each enhancement category and artifact sub type (N= 33).

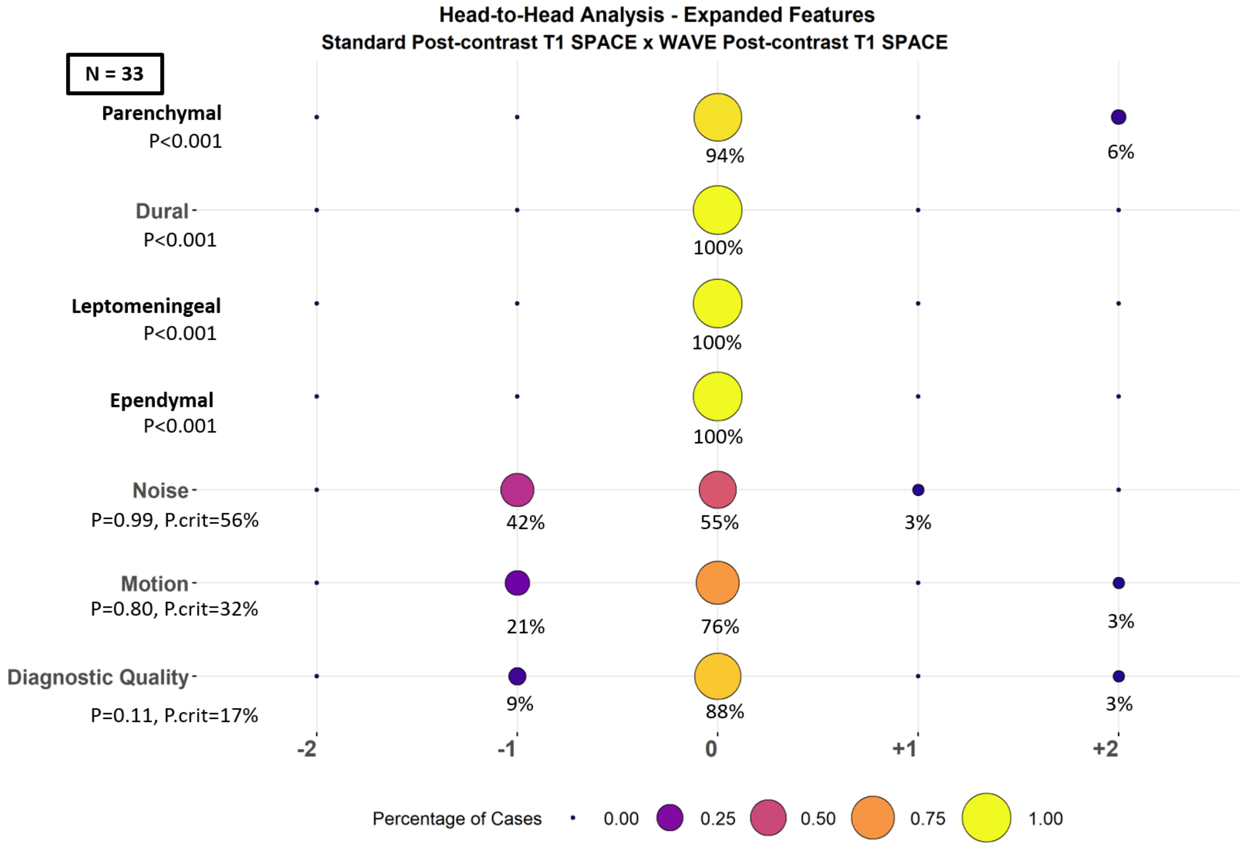

Supplement: Supplementary file 2 [file Image_1.PDF]
